# Fulminant Panuveitis following Iris Suture Fixation of Posterior Chamber Intraocular Lens

**DOI:** 10.1155/2013/910342

**Published:** 2013-02-12

**Authors:** Ahmad M. Mansour, Shady T. Awwad

**Affiliations:** ^1^Department of Ophthalmology, American University of Beirut, P.O. Box 1136044, Beirut, Lebanon; ^2^Department of Ophthalmology, Rafic Hariri University Hospital, P.O. Box 11-5344, Beirut, Lebanon

## Abstract

We present a case of fulminant panuveitis following iris suture fixation of a posterior chamber intraocular lens. We hypothesize that the zonular dehiscence allowed the inflammatory cells in the anterior compartment to gain access to the posterior segment mimicking endophthalmitis or toxic anterior segment syndrome. Also certain bulky lens designs, like the current Rayner hydrophilic acrylic lens, are difficult to manipulate and hold in the optic capture position, and hence the iris fixation of these lenses can be traumatic and lengthy. It is advised to exchange such lenses with 3-piece intraocular lenses that are easy to fixate.

## 1. Introduction

The concept of iris suture fixation for posterior chamber intraocular lenses dates back to 1976, when Malcolm McCannel, M.D., described his trans-corneal suture technique to stabilize subluxated posterior intraocular lenses. Since then, iris suture fixation has become a well-established effective means for stabilizing posterior chamber lenses in the lack of adequate capsular support [1–8]. The technique consisted of a McCannel 10-0 polypropylene suture which was used to fixate the haptics to the iris using the Siepser sliding knot [5]. In a series of 46 patients [6], the main complications of iris suture fixation included transient low-grade uveitis in 3 (6.5%), transient pigment dispersion in 3 (6.5%), and intraocular lens dislocation in 2 (4.3%). Additionally in a second series of 17 eyes of 9 children [7], other complications of iris suture fixation included hyphema in 1 case and sterile endophthalmitis in another case. A case of severe uveitis and severe visual loss after iris suture fixation is described.

## 2. Case Report

**Figure 1:**
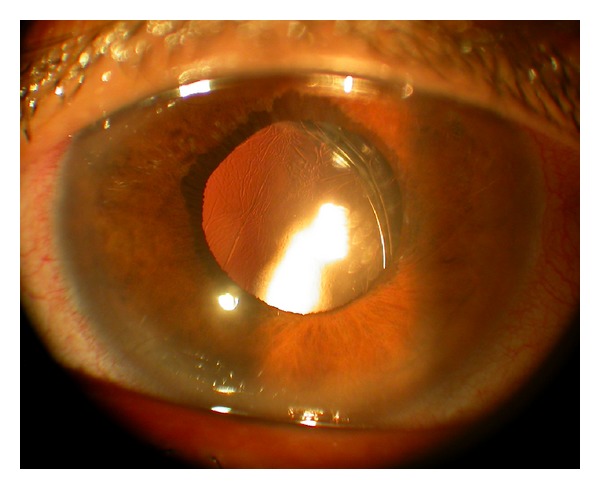
Preoperative anterior segment photograph of the left eye shows mild subluxation of Rayner Superflex hydrophilic acrylic intraocular lens with uncorrected visual acuity of 6/9.

**Figure 2:**
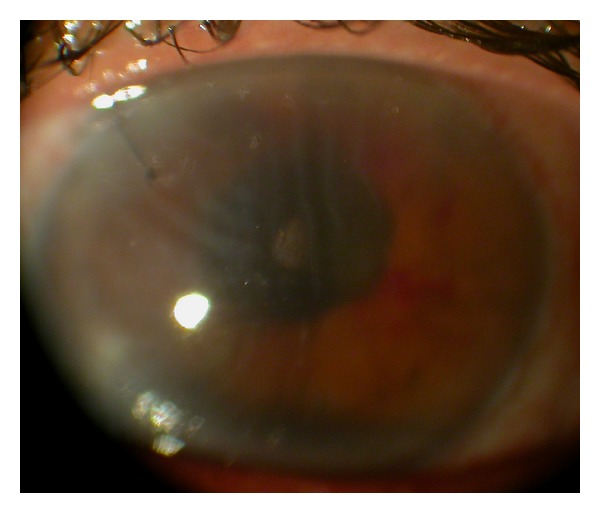
Ten hours after surgery, the left eye has fibrinous iritis with visual acuity of finger counting at 10 cm. The cornea has mild stromal edema and Descemet membrane folds. The pupil is mid-constricted.

**Figure 3:**
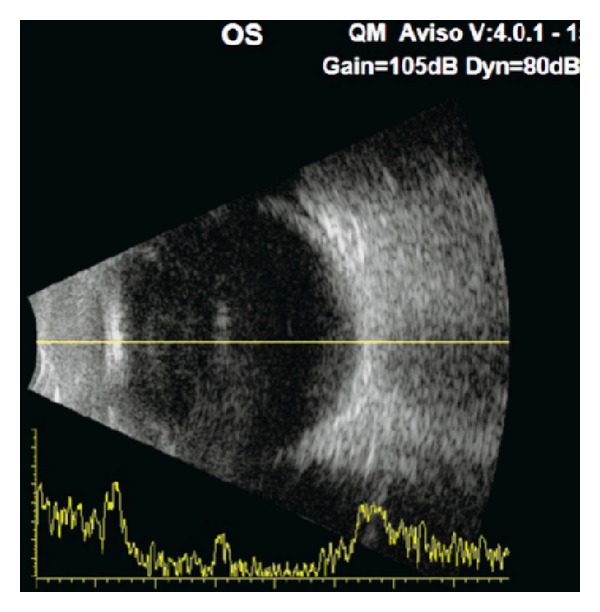
B-scan demonstrates abnormal irregular medium reflections in the midvitreous cavity of the left eye.

**Figure 4:**
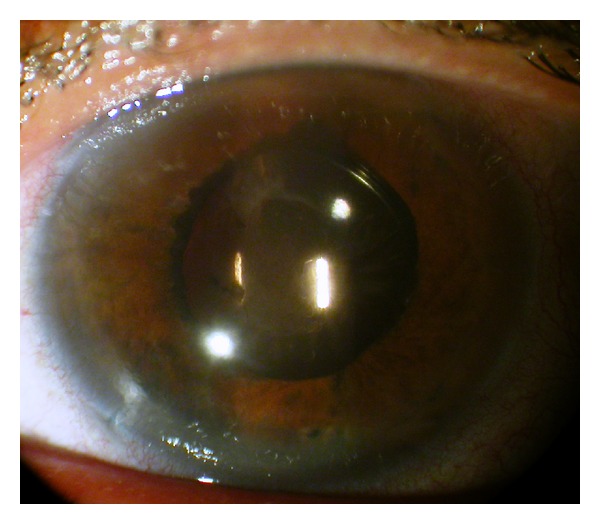
One week postoperatively, the anterior segment of the left eye appears quiet with 6/21 visual acuity.

This 46-year-old Iraqi lady had prior anterior chamber intraocular lens implantation for familial lens subluxation and previous pars plana vitrectomy for retinal detachment ending with poor vision in the right eye. The left eye underwent scleral buckle for rhegmatogenous retinal detachment with findings of severe scleral thinning. Subsequently she had phacoemulsification with hydrophilic acrylic intraocular lens (Superflex, Rayner Intraocular Lenses Ltd, East Sussex, UK; 6.25 mm optic and 12.5 mm overall length) implantation in the bag with zonular dehiscence. Visual acuity was 6/9 in the left eye with mild decentration (Figure 1). The patient was referred for scleral fixation of the lens. Because of scleral thinning and history of retinal detachment, we proceeded with iris suture fixation. Two surgeons were working simultaneously through several keratome incisions, and it was necessary to place repeated viscoelastic (2.4 mL of sodium hyaluronate) to avoid corneal touch by the lens. The lens was fixated by the first surgeon using intraocular forceps used in vitreous surgery in the right hand and a Sinskey hook to maintain optic capture with the left hand. The second surgeon performed iris suture fixation superiorly and inferiorly. The inferior suture led to lens tilt that was not relieved by gentle iris massage around the suture and thereafter that suture was removed. Surgery was done at night and lasted 75 minutes. Ten hours postoperatively, the patient had finger counting at 10 cm with fibrinous panuveitis (Figure 2) and dense echoes on B-scan (Figure 3). The possibility of endophthalmitis was discussed with the patient who declined immediate aqueous paracentesis for culture purposes. She was therefore followed up several times daily initially. Intensive oral and topical corticosteroid therapy with topical nonsteroidal anti-inflammatory drops controlled the inflammation as early as 24 hours after surgery. Visual acuity improved to 6/21 one week postoperatively with minimal iritis (Figure 4). Oral and topical corticosteroids (dexamethasone drop every half hour and 100 mg prednisone daily) were substituted with loteprednol etabonate because of severe ocular hypertension (intraocular pressure of 38 mm Hg). Intraocular pressure dropped to 12 mm Hg and visual acuity dropped to 6/24 from residual mild uveitis on discharge 2 weeks after surgery. The patient travelled to Iraq on loteprednol and non-steroidal anti-inflammatory drop. She reported a gradual recovery of preoperative vision 5 weeks after surgery. Rheumatologic workup was negative (including rheumatology consult, antinuclear antibodies, and rheumatoid arthritis latex test).

## 3. Discussion

In the current patient, scleral fixation could not be performed due to scleral ectasia noted during scleral buckle making scleral flap more technically difficult in addition to the risk of retinal redetachment and expulsive hemorrhage [8]. Another alternative is to perform intraocular lens exchange with a 3-piece foldable intraocular lens that can be easily fixated to the iris. Also the findings of severe fibrinous reaction several hours after surgery led us to suspect early endophthalmitis. The absence of hypopyon, ocular pain, and lid edema favored sterile endophthalmitis in the current case and as described by Dureau et al. [7] after iris suture fixation. Toxic anterior segment syndrome was also included in the differential diagnosis. In a series of 126 patients (137 eyes) who were implanted with a posterior iris-claw aphakic intraocular lens, endophthalmitis, toxic anterior segment syndrome, and chronic uveitis occurred in 1 patient each [9]. Also, van Philips [10] described toxic anterior segment syndrome in 4 eyes of 3 patients who had foldable Artiflex iris-fixated phakic intraocular lens implantation. The following features were not in favor of toxic anterior segment syndrome: maximum use of disposable instrument, little use of balanced salt solution without phenylephrine, presence of vitritis, and absence of corneal stromal edema. We opted for very close observation and intense corticosteroid therapy orally and topically along with non-steroidal anti-inflammatory drops. We hypothesize that the zonular dehiscence allowed the inflammatory cells in the anterior compartment to gain access to the posterior segment [11–13], in addition to the presence of choroiditis from surgical trauma, hence the presence of dense vitreous echoes by ultrasonography.

The Rayner hydrophilic acrylic intraocular lens is bulky, slippery, and difficult to hold in the optic capture position and also it is difficult to have an imprint of the haptic to the posterior iris. In this respect, 2 surgeons performed the procedure: the first stabilizing the optic in the anterior segment while the second surgeon (well-experienced in this technique) passing the McCannel suture from limbus to limbus through the invisible haptic. Usually the temporary optic capture stabilizes by itself the three-piece, foldable acrylic intraocular lens and by the same token facilitates placing the McCannel sutures [2–6].

Iris suturing of intraocular lens may be very difficult to achieve in some intraocular designs (e.g., Rayner Superflex one-piece acrylic lenses). Clinicians need to minimize iris manipulation, and if such tissue trauma occurs, aggressive anti-inflammatory therapy needs to be instituted intraoperatively and postoperatively.
